# Functional Polymorphism of the CK2α Intronless Gene Plays Oncogenic Roles in Lung Cancer

**DOI:** 10.1371/journal.pone.0011418

**Published:** 2010-07-02

**Authors:** Ming-Szu Hung, Yu-Ching Lin, Jian-Hua Mao, Il-Jin Kim, Zhidong Xu, Cheng-Ta Yang, David M. Jablons, Liang You

**Affiliations:** 1 Thoracic Oncology Laboratory, Department of Surgery, Comprehensive Cancer Center, University of California San Francisco, San Francisco, California, United States of America; 2 Division of Pulmonary and Critical Care Medicine, Chang Gung Memorial Hospital, Chiayi, Taiwan; 3 Graduate Institute of Clinical Medical Sciences, College of Medicine, Chang Gung University, Taoyuan, Taiwan; 4 Life Sciences Division, Lawrence Berkeley National Laboratory, University of California, Berkeley, California, United States of America; 5 Department of Respiratory Care, College of Medicine, Chang Gung University, Taoyuan, Taiwan; University of Hong Kong, Hong Kong

## Abstract

Protein kinase CK2 is frequently up-regulated in human cancers, although the mechanism of CK2 activation in cancer remains unknown. In this study, we investigated the role of the CK2α intronless gene (*CSNK2A1P*, a presumed CK2α pseudogene) in the pathogenesis of human cancers. We found evidence of amplification and over-expression of the *CSNK2A1P* gene in non- small cell lung cancer and leukemia cell lines and 25% of the lung cancer tissues studied. The mRNA expression levels correlated with the copy numbers of the *CSNK2A1P* gene. We also identified a novel polymorphic variant (398T/C, I133T) of the *CSNK2A1P* gene and showed that the 398T allele is selectively amplified over the 398C allele in 101 non-small cell lung cancer tissue samples compared to those in 48 normal controls (*p* = 0.013<0.05). We show for the first time CSNK2A1P protein expression in transfected human embryonic kidney 293T and mouse embryonic fibroblast NIH-3T3 cell lines. Both alleles are transforming in these cell lines, and the 398T allele appears to be more transforming than the 398C allele. Moreover, the 398T allele degrades PML tumor suppressor protein more efficiently than the 398C allele and shows a relatively stronger binding to PML. Knockdown of the *CSNK2A1P* gene expression with specific siRNA increased the PML protein level in lung cancer cells. We report, for the first time, that the *CSNK2A1P* gene is a functional proto-oncogene in human cancers and its functional polymorphism appears to degrade PML differentially in cancer cells. These results are consistent with an important role for the 398T allele of the *CSNK2A1P* in human lung cancer susceptibility.

## Introduction

Lung cancer is the leading cause of death from cancer in the United States [1]. Some of the somatic events involved in lung cancer have been well characterized, but some of them remain unknown [2], [3]. Protein kinase CK2 (formerly known as casein kinase II) is a serine/threonine protein kinase that phosphorylates more than 300 proteins [4]. It degrades tumor suppressor proteins such as PML [5] and promotes the activity and stability of oncogenic proteins such as AKT [6]. For instance, CK2 promotes PML degradation and CK2 kinase activity is inversely correlated with PML protein levels in human lung cancer [5]. Recently, multiple myeloma cell survival was shown to rely on the high activity of protein kinase CK2 [7]. CK2 also affects several cell signaling pathways, including PI3K, NFkB, and Wnt pathways [8].

The level of CK2α expression is tightly regulated in normal cells [9], and increased CK2α level and activity has consistently been observed in a variety of human cancers [10]. For instance, the high-level and/or nuclear localization of CK2α is a marker of poor prognosis for patients with acute myeloid leukemia, prostate cancer, and squamous cell lung cancer [10]. CK2α is the catalytic subunit of protein kinase CK2 and CK2α has been reported to be coded by the *CSNK2A1* gene on chromosome 20p13 [11]. The *CSNK2A1* gene can serve as an oncogene and its dysregulated expression can induce mammary tumors and lymphomas in transgenic mice [12], [13]. Although the activity of the *CSNK2A1* gene has been shown to be elevated in human cancers, no solid genetic or epigenetic evidence is available regarding the cause of the high-activity of CK2α in cancer cells [10]. The CK2α intronless gene (also known as CK2α “pseudogene”), *CSNK2A1P*, is located on chromosome 11p15.3 and its sequence is highly homologous (99% identity) to the *CSNK2A1* cDNA sequence [14]. Although the *CSNK2A1P* gene is reportedly expressed in a megakaryocytic cell line [15], its role in cancer cell development remains unknown. We therefore investigated the amplification and expression of the *CSNK2A1P* gene in lung cancer and leukemia cell lines and lung cancer tissues.

## Results

### Over-expression and amplification of the *CSNK2A1P* gene in cancer cells and lung cancer tissues

**Figure 1:**
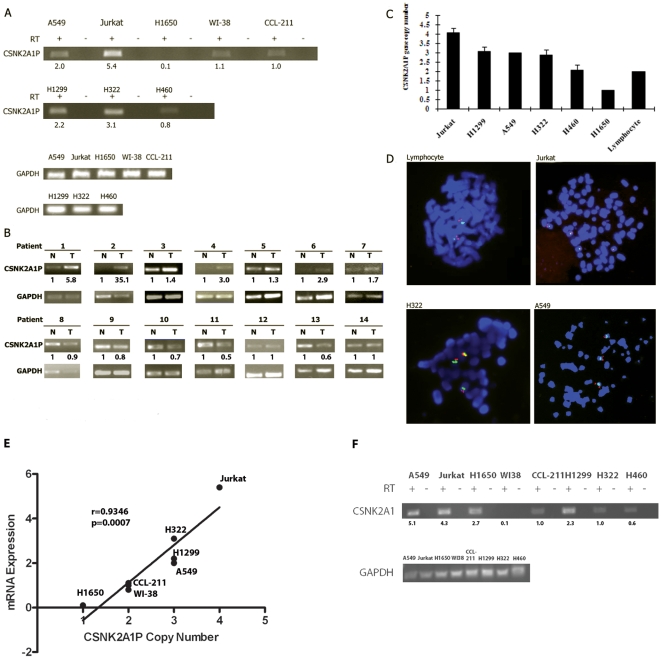
*CSNK2A1P* gene overexpression amplification in human cancer cell lines and primary tumors. (A) Semi-quantitative RT-PCR using CSNK2A1P-specific primers shows the CSNK2A1P mRNA expression level is consistent with the copy numbers detected by FISH (3 for H1299, 4 for Jurkat, 3 for H322 and A549, 2 for WI-38 and CCL-211). (B) Semi-quantitative RT-PCR using CSNK2A1P-specific primers shows the CSNK2A1P mRNA is overexpressed in seven (patient 1 to 7) out of 29 (∼25%) lung tumors as compared to matched normal adjacent tissue. Patient 8 to 14 represents samples without overexpressed CSNK2A1P mRNA. Other 14 samples with similar results are not shown. (C) The *CSNK2A1P* gene is amplified in the human T cell leukemia cell line Jurkat, and in lung cancer cell lines H1299, A549, and H322. (D) Representative pictures of FISH study results in metaphases of normal lymphocyte, Jurkat, H322 and A549 cell lines. The *CSNK2A1P* gene was labeled with Cy3 (in red). Chromosome 11 centimeter probe was labeled with FITC (in green). (E) Correlation of the copy number and mRNA expression the *CSNK2A1P* gene was calculated using pearson correlation. (F) Semi-quantitative RT-PCR using CSNK2A1-specific primers shows the CSNK2A1 mRNA expression level in normal and cancer cell lines mentioned above. All semi-quantitative RT-PCR experiments were repeated three times with similar results.

We focused on the *CSNK2A1P* gene because we initially found it was minimally expressed in normal cells, but was predominantly expressed in several types of human cancer cell lines and tumor tissues, including the human T cell leukemia cell line Jurkat, which is known for its high CK2 activity. By semi-quantitative RT-PCR, we showed that the *CSNK2A1P* gene is also over-expressed in three NSCLC lines: H1299, H322, and A549, but minimally expressed in normal cells and two lung cancer cell lines H1650 and H460 (Fig. 1A). Furthermore, *CSNK2A1P* mRNA was overexpressed in lung tumor tissue from seven out of 29 (∼25%) tumor samples as compared to matched control tissues (Fig. 1B). Moreover, FISH analysis on the same human cancer cell lines provided solid evidence of the amplification of the *CSNK2A1P* gene located on chromosome 11p15.3 (Fig. 1D). For example, four copies of the *CSNK2A1P* gene were found in the human T cell leukemia cell line Jurkat, and three copies in the lung cancer cell lines H1299, A549, and H322. In contrast, normal lymphocytes and H460 cell line had diploid copies of the *CSNK2A1P* gene (Fig. 1C and D). Importantly, mRNA expression levels correlated with copy numbers of the *CSNK2A1P* gene in these human cancer cell lines (n = 8; pearson's γ = 0.9346; *p* = 0.0007) (Fig. 1 E). We further performed semi-quantitative RT-PCR to evaluate the mRNA expression of the *CSNK2A1* gene with specific primers. Overexpression of the *CSNK2A1* gene was also noted in above cancer cell lines (Fig. 1F), suggesting that in addition to the *CSNK2A1* gene, the *CSNK2A1P* gene also plays important roles in the pathogenesis of lung cancer. Alignment of the primers used for semi-quantitative RT-PCR of the *CSNK2A1* and *CSNK2A1P genes* were shown in supplementary data (Fig. S1).

### Allele-specific amplification of the *CSNK2A1P* gene in lung cancer

**Figure 2:**
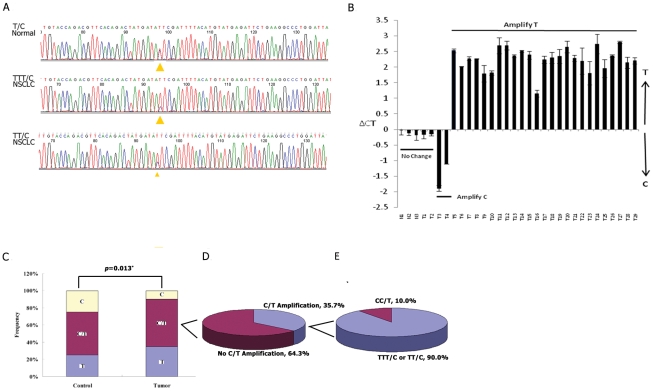
Allele-specific amplification of *CSNK2A1P* gene in lung cancer. (A) Allele-specific amplification of the 398T allele (TTT/C or TT/C) is found in some non-small cell lung cancer tissue samples. The T above the arrowhead (nucleotide 398T, I133,) on the right is the indication of the wild-type gene. (B) Amplification of *CSNK2A1P* alleles in lung tumors was validated by quantitative allele specific single polymorphism (SNP) analysis. The ratio between the 398T and 398C alleles in heterozygous lung tumors was determined by TaqMan analysis using allele-specific probes and is expressed as differences in CT levels (cycles) with positive or negative ΔCT values indicating amplification of the 398T or 398C allele, respectively. Of 56 samples typed, 2 showed amplification of the 398C allele greater than 0.6 CTs (samples T3–4) and 18 showed amplification of the 398T allele (samples T5–23). Thirty-six samples showed no amplification (represented by T1–2). Normal controls samples showed no amplification (N1–3). The normalized means and standard deviations of three independent experiments are shown. (C, D and E) Allele-specific amplification of the 398T allele significantly increases in non-small cell lung cancer tissue samples. Normal: human adult normal genomic DNA. Tumor: no-small cell lung cancer tissues. * denotes p<0.05 (Chi-square test).

To determine whether there are any mutations of *CSNK2A1P* in human lung cancers, we sequenced the open reading frame of the *CSNK2A1P* gene. Although we did not find any mutations, we discovered a novel polymorphism within the kinase domain (398T/C, which leads to amino acid change I133T, Fig. 2A) in 18 cancer cell lines (Table 1) and 101 primary lung cancer tissue samples (Table 2). The specific amino acid change is predicted to affect the protein function when the sorting intolerant from tolerant (SIFT) method is used [16]. Interestingly, this polymorphism appeared to be evenly distributed in normal tissue, but in tumors, was significantly more frequent at the 398T allele (Fig. 2C) (Chi-Square test, p<0.05). Furthermore, of the 56 heterozygous lung cancer tissues examined with respect to this polymorphism (Fig. 2D), 20 (35.7%) samples showed amplification in this region, and in 18 (90%) of those 20 samples, the 398T allele was selectively amplified (Fig. 2E). This selective amplification suggests that the 398T allele might provide a growth advantage over the 398C allele. To further validate the results from DNA sequencing, we investigated the allele-specific amplification of the 398T allele of *CSNK2A1P* (encoding the Ile133 variant) in human tumors using a quantitative single nucleotide polymorphism (SNP) analysis, i.e., TaqMan-based allelic discrimination assay. The allelic discrimination data is consistent with the sequencing data (Fig. 2B). Of the 101 lung tumor samples typed, 56 were heterozygous with respect to the 398C→T polymorphism, and we analyzed these 56 samples for allele-specific amplification of the 398C→T polymorphism of *CSNK2A1P* by TaqMan analysis (Fig. 2B). Twenty samples showed allelic imbalance between the two alleles, 2 samples showed gain of the 398C allele (encoding Thr133) and 18 samples showed gain of the 398T allele (encoding Ile133). These results show statistically significant allele-specific amplification of the 398T allele (*P*<0.05, Chi-Square test), providing additional evidence for the role of this allele in human lung cancer. These results prompted us to probe more deeply into the role of the variant *CSNK2A1P* forms in tumor development.

**Table 1:** DNA sequencing results of the 398 allele in cancer cell lines.

Cell line type and name		398T	398T/C	398C
**Lung cancer**	**H1299**		**+**	
**Lung cancer**	**A549**		**+**	
**Lung cancer**	**A427**	**+**		
**Lung cancer**	**H322**			**+**
**Lung cancer**	**H358**			**+**
**Lung cancer**	**H441**	**+**		
**Lung cancer**	**H460**	**+**		
**Lung cancer**	**H838**	**+**		
**Lung cancer**	**H1650**			**+**
**Lung cancer**	**H1703**	**+**		
**Lung cancer**	**H1975**			**+**
**T cell leukemia**	**Jurkat**	**+**		
**Colon cancer**	**HCT116**			**+**
**Cervical cancer**	**Hela**	**+**		
**Mesothelioma**	**MS-1**	**+**		
**Mesothelioma**	**H28**	**+**		
**Mesothelioma**	**H2052**	**+**		
**Mesothelioma**	**H290**	**+**		

**Table 2:** DNA sequencing results of the 398 allele in 101 lung cancer tissues samples and genomic DNA from 48 normal adults.

Sequencing results	Lung cancer samplesSample numbers (%)	Normal genomic DNASample numbers (%)
CC/T	**2 (2%)**	0 (0%)
TT/C or TTT/C	**18 (17.8%)**	0 (0%)
T	35 (34.6%)	12 (25%)
C/T or T/C	36 (35.6%)	24 (50%)
C	10 (10%)	12 (25%)

### Differential transforming activity of the two *CSNK2A1P* alleles

**Figure 3:**
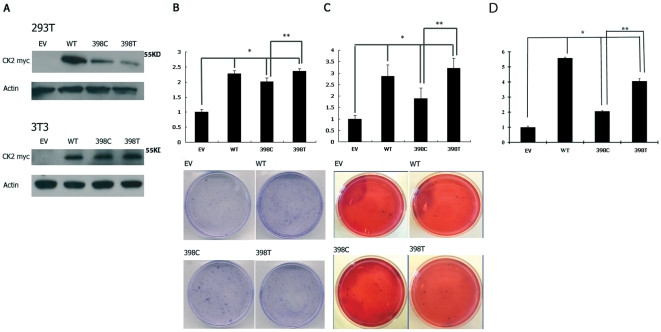
Differential transforming activity of the two *CSNK2A1P* alleles. (A) The *CSNK2A1* and *CSNK2A1P* genes were transiently transfected into 293T and NIH3T3 cells using Lipofectamine 2000 Reagent according to manufacture's protocol. 72 hours after transfection, cells were harvest and total cellular proteins were extracted. Western blot analysis was used to confirm the protein expression in both alleles of the *CSNK2A1P* gene. Anti-Myc tag antibody was used to detect the CSNK2A1P-Myc tag fusion proteins. (B) Colony formation assay. Transfection of *CSNK2A1P* gene in NIH3T3 cells results in enhanced anchorage-dependent growth, compared to the empty vector control. The colony numbers of NIH3T3-CSNK2A1 and NIH3T3-CSNK2A1P cells are dramatically higher than those of the NIH3T3-EV cells. (C) Soft agar assay. Stable transfection of *CSNK2A1P* genes in NIH3T3 cells results in enhanced anchorage-independent growth. Both the NIH3T3-CSNK2A1 and NIH3T3-CSNK2A1P cells produced significantly more colonies than the NIH3T3-EV cells did (*p<0.05, t-test). Of the two alleles of the *CSNK2A1P* gene, the 398T allele formed more colonies than the 398C allele did (**p<0.05, t-test). Relative colony formation in both cell lines is expressed as percentage normalized to empty-vector-transfected control group and shown as bar ± standard deviation in three independent experiments. (D) Kinase assay of the expressed CSNK2A1 and CSNK2A1P proteins in NIH3T3 cells. The expressed proteins were immune-precipitated with Anti-Myc tag antibody and kinase assay was performed. Both the NIH3T3-CSNK2A1 and NIH3T3-CSNK2A1P cells had significantly higher kinase activities than the NIH3T3-EV cells did (*p<0.05, t-test). Of the two alleles of the *CSNK2A1P* gene, the 398T allele cells had significantly higher kinase activity than the 398C allele did (**p<0.05, t-test). Relative kinase activity is expressed as percentage normalized to empty-vector-transfected control group and shown as bar ± standard deviation in three independent experiments. EV: empty vector. Wt: CSNK2A1. 398C: CSNK2A1P (I133) 398T: CSNK2A1P (I133T).

To test the functional significance of the 398T→C polymorphism in the *CSNK2A1P* gene, we cloned the *CSNK2A1* and *CSNK2A1P* genes into pcDNA 3.1/myc-His vector and carried out a series of cell growth assays using NIH3T3 cells. In this study, these vectors were transiently transfected into 293T and NIH3T3 cells and western analysis was used to confirm the protein expression of both alleles of the *CSNK2A1P* gene (Fig. 3A). The results show, for the first time, that the expression of *CSNK2A1P* can produce its proteins.

In the colony formation assay, transfection of *CSNK2A1P* genes in NIH3T3 (NIH3T3-CSNK2A1P) cells results in enhanced anchorage-dependent growth, compared to the empty vector control (NIH3T3-EV). In addition, we generated NIH3T3 cells with CSNK2A1 (NIH3T3-CSNK2A1) as a positive control. The colony numbers of NIH3T3-CSNK2A1 and NIH3T3-CSNK2A1P cells were dramatically higher than those of the NIH3T3-EV cells (Fig. 3B). Transfection of the *CSNK2A1P* gene in NIH3T3 cells also resulted in enhanced anchorage-independent growth. When soft agar colony formation assay results were compared, we found that stable transfection of *CSNK2A1P* genes in NIH3T3 cells resulted in enhanced anchorage-independent growth (Figure 3C). Both the NIH3T3-CSNK2A1 and NIH3T3-CSNK2A1P cells produced more colonies than the NIH3T3-EV cells did. Compared to the NIH3T3-EV cells, NIH3T3-CSNK2A1 and NIH3T3-CSNK2A1P cells produced colonies that were mostly bigger and had spread-out shapes. When the two alleles of the *CSNK2A1P* genes were compared, we found that the 398T allele formed more colonies than the 398C allele did. A kinase assay of the expressed proteins was also performed (Figure 3D). The 398T gene product has a higher kinase activity than the 398C gene product does. The results from the kinase assay are consistent with the colony formation and soft agar data.

### Functional polymorphism of the *CSNK2A1P* genes on the degradation of PML tumor suppressor protein

**Figure 4:**
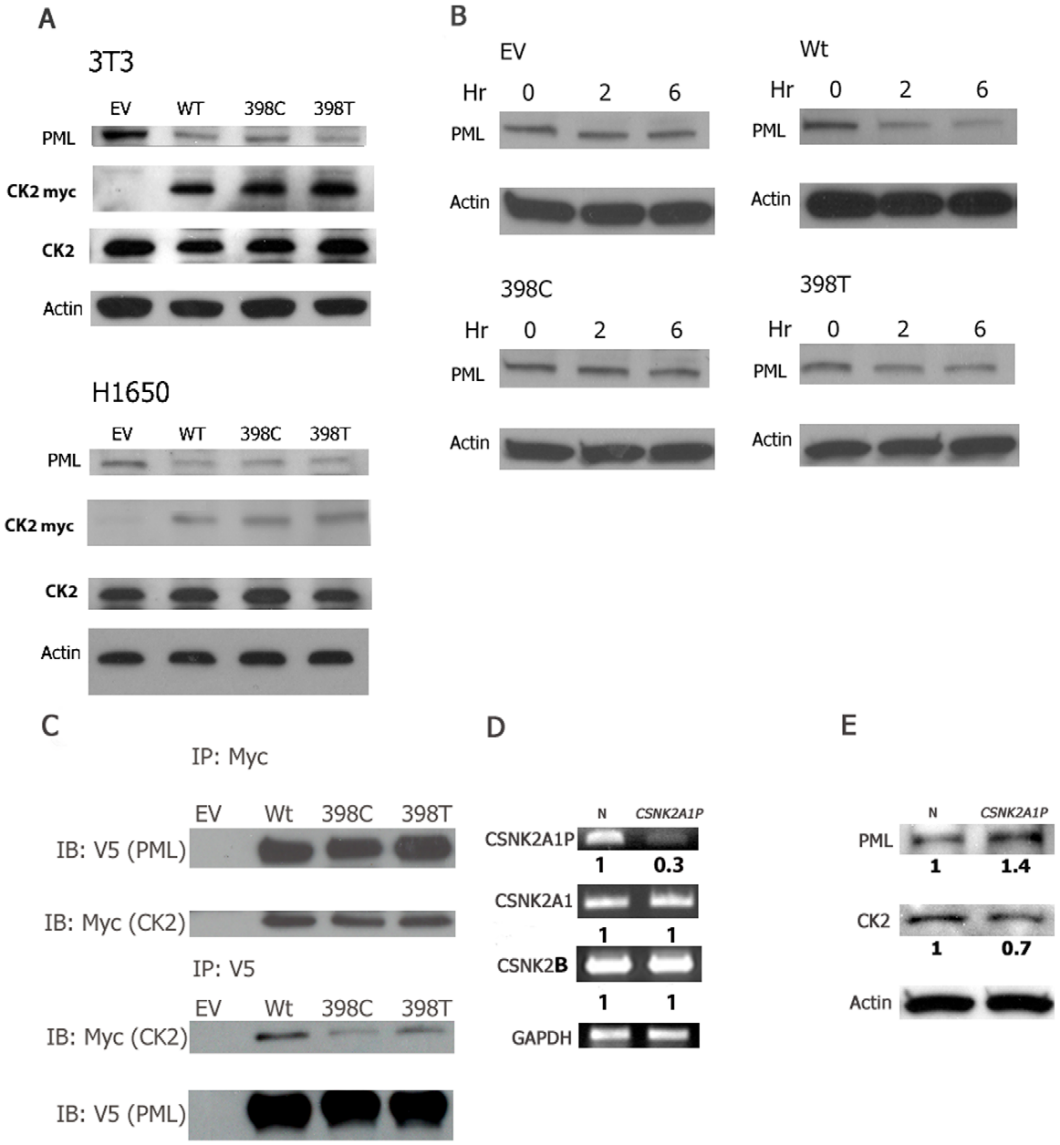
Functional polymorphism of the *CSNK2A1P* genes on the degradation of PML tumor suppressor protein. (A) The PML protein decreases in NIH3T3 and H1650 stable cell lines that were transfected by the *CSNK2A1* and *CSNK2A1P* genes. The endogenous CSNK2A1 protein and overexpressed CSNK2A1 and CSNK2A1P proteins were also shown. (B) Degradation of the PML protein is more prominent in NIH3T3 stable cell lines transfected with CSNK2A1 and CSNK2A1P (I133). Hr: hours after cycloheximide treatment. (C) 293T cells were co-transfected with PML-V5 and Myc-tagged CSNK2A1 or CSNK2A1P constructs. CSNK2A1 and CSNK2A1P (I133) show stronger binding to the PML protein in reciprocal immunoprecipitation assays. EV: empty vector. Wt: CSNK2A1. 398T: CSNK2A1P (I133) 398C: CSNK2A1P (I133T). (D) The expression of the *CSNK2A1P* gene decreases in the H1299 lung cancer cell line after transfection with the *CSNK2A1P* gene specific siRNA. No decrease in the expression of the *CSNK2A1* and *CSNK2B* genes were noted. (E) The CK2α protein level decreases and the PML protein level increases in the H1299 lung cancer cell line after transfection with the *CSNK2A1P* gene specific siRNA. N: negative control siRNA. CSNK2A1P: *CSNK2A1P* siRNA. The band densities are normalized to the negative siRNA transfected group using actin as an internal control. All experiments were repeated three times with similar results.

To determine the potential mechanism by which the 398T allele is preferentially amplified in lung cancer samples, we studied the effects of the two alleles of the *CSNK2A1P* gene on tumor suppressor PML protein. The *CSNK2A1P* genes were stably transfected into NIH3T3 and NSCLC H1650 cell lines. Western blot analysis showed a reduced PML protein level in the cells transfected with the *CSNK2A1P* genes. The CSNK2A1P 398T allele, similar to wild-type CSNK2A1, decreased PML protein levels more than the 398C allele did (Fig. 4A). Second, we determined whether the half-life of PML is differentially regulated by the two alleles. These two cell lines transfected with the *CSNK2A1P* gene were treated with cycloheximide for 0, 2, and 6 hours, and the endogenous PML protein levels were examined. The 398T allele decreased the half-life of PML more effectively than the 398C allele did (Fig. 4B). Third, we used co-immunoprecipitation to show the direct and preferential binding between the 398T protein and PML protein (Fig. 4C).

To further address the question of whether the CSNK2A1P mRNA is fully translated and degrades PML in cancer cells, we knocked down the *CSNK2A1P* gene in H1299 lung cancer cell lines with siRNA specific to the *CSNK2A1P* gene. We chose the H1299 lung cancer cell line because our study (Figure 1A and C) showed that the *CSNK2A1P* gene is amplified and overexpressed in this cell line. The *CSNK2A1P* gene specific siRNA caused a greater than 70% decrease in the expression of the *CSNK2A1P* gene and no decrease in the expression of the *CSNK2A1* and *CSNK2B* genes (Fig. 4D). In accordance with decreased expression of the *CSNK2A1P* gene, the total CK2α protein decreased and the PML protein increased in the cells treated with *CSNK2A1P* siRNAs compared to negative siRNA transfected controls (Fig. 4E).

## Discussion

In this study, we have provided, to our knowledge, the first evidence to show that the *CSNK2A1P* gene is a functional proto-oncogene in human cancers. To support our hypothesis that *CSNK2A1P* gene is a functional proto-oncogene, rather than a “pseudogene”, we have done extensive DNA, mRNA, protein and siRNA analysis. The results of this analysis provide the first evidence that the mRNA expression level correlates with the copy number of the *CSNK2A1P* gene in several human cancer cell lines. Furthermore, siRNA knock down of the *CSNK2A1P* gene decreased total CK2α protein level in cancer cells, indicating that the *CSNK2A1P* gene is fully translated in cancer cells. Thus, the amplification of the *CSNK2A1P* gene may play an oncogenic role in these human cancer cell lines. On the basis of our results, we propose that two functional genes may exist in the human CK2α family, *CSNK2A1*, which locates on the chromosome 20p13, and *CSNK2A1P*, which locates on the chromosome 11p15.3.

We also found a novel polymorphism within the kinase domain (398T/C, which leads to amino acid change I133T, Figure 2a) in 101 primary tumors. This polymorphism appears to be evenly distributed in normal tissue, but in tumors, is significantly more frequent in the 398T allele (Table 2, Figure 2). The 398T allele was selectively amplified in eighteen of the 101 lung cancer tissues, suggesting it might provide a growth advantage over the 398C allele. The 398C allele amplification was only found in two of the 101 lung cancer tissues, suggesting that it might be a random event. These intriguing genetic results prompted us to probe more deeply into the role of the variant *CSNK2A1P* forms in tumor development. Doing so yielded two important findings, first, that the 398T allele is more transforming than the 398C allele, and second, that the 398T allele degrades tumor suppressor PML protein more efficiently than the 398C allele. For instance, the 398T allele showed significantly higher transforming activity in both colony formation and soft agar assays than the 398C allele (Fig. 3B and C). The data of the kinase assay showed that the 398T gene product has higher kinase activity than the 398C gene product (Fig. 3D), suggesting that the 398T allele is a more transforming allele than the 398C allele. In addition, these data also suggest that the transforming ability of the 398T allele of the *CSNK2A1P* pseudogene is similar to that of the normal *CSNK2A1* gene product, so the more active allele of the pseudogene appears to have activity comparable to the regular *CSNK2A1* gene. To date, there is no genetic or epigenetic evidence on the activation of the CSNK2A1 gene in human cancer. Thus our study provides the first evidence that the *CSNK2A1P* 398T allele, which is similar to the *CSNK2A1* gene, is amplified in human lung cancer tissues.

The PML gene was originally identified at the breakpoint of t(15;17) translocation in acute promyelocytic leukemia [17] and was proved to be a tumor suppressor gene [18]. Loss of PML has been correlated with poor clinical outcome in a variety of human cancers, including lung cancer, supporting its tumor suppressor function [5], [19]. PML promotes the dephosphorylation of pRb and regulates cell fate in the developing neocortex [20]. CK2 regulates ubiquitin-mediated degradation of PML in human lung cancer cell lines [5], [21]. This may be one the potential mechanisms by which the 398T allele is preferentially amplified in lung cancer samples. This genetic polymorphism is probably a useful marker to detect the amplification of the CK2 intronless gene, because monoallelic amplification is believed to be responsible for gene amplification in cancer [22]. This allele-specific amplification also implies that cancer cells may be addicted to the oncogenic CK2α [23]. In short, the amplification of the 398T allele of the *CSNK2A1P* gene may contribute, at least in part, to the pathogenesis of lung cancer.

The *CSNK2A1P* gene is a processed pseudogene located on the chromosome 11p15.3 and is supposed to be formed by retrotransposition, and characterized by the absence of introns, the presence of flanking directs repeats, and the 3′ polyadenylation tail [15]. However, it has a strong promoter upstream from the initiation codon (14, 15). The expression of the *CSNK2A1P* gene is potentially more tightly regulated than the *CSNK2A1* is. For instance, the promoter region of the *CSNK2A1P* gene contains two TATA boxes and a CAAT box, while the upstream sequence of *CSNK2A1* displays housekeeping gene characteristics, e.g., high GC content, the presence of several GC boxes and the lack TATA box (14, 15). Moreover, several important transcription factor-binding sites (e.g, CEBP-, GATA-, and SMAD-binding sites) were predicted within the 5′ region (1 Kb) from the *CSNK2A1P* starting codon, suggesting that the *CSNK2A1P* gene may potentially be induced or repressed by several master regulators of developmental pathways. The amplification of the *CSNK2A1P* gene could result in the overexpression of CSNK2A1P protein in cancer cells. It may also be possible that the *CSNK2A1* gene is somehow indirectly regulated by the expression of the *CSNK2A1P* gene. Future studies are needed to uncover the mechanism through which the *CSNK2A1P* and the *CSNK2A1* genes are regulated. Until now, around 10,000 processed pseudogenes [24] have been characterized in the human genome, and some of these genes have been reported to be expressed in cancer cells. For instance, the oncogenic *CRIPTO3* pseudogene is expressed in colon, breast and lung cancers [25], the human homologue of vaccinia virus H1 phosphotase gene clone 5 (*hVH-5*) pseudogene is expressed in breast cancer cell lines [26], and the *rac1* pseudogene is expressed in brain tumors [27]. Taken together with our results, this suggests a potential oncogenic role of the presumed pseudogenes in some human cancers.

This study had some limitations. We demonstrated the correlation between *CSNK2A1P* gene and PML protein stability in only two lung cancer cell lines in vitro. Specimens from only 101 lung cancer patients were analyzed, 56 of which were heterozygous with respect to this polymorphism and could be used for the analysis. In future studies, it will be important to include additional cancer cell lines and more cancer tissues.

Our results provide genetic evidences for activation of the intronless CK2α gene in human cancer. Although CK2 was previously known to be a key player in cancer, its mechanism of activation in cancer was not known [10], [28]. Because of this lack of knowledge about the mechanism of CK2 activation and its constitutive activity, CK2 has been mostly neglected as a key target for anti-cancer drugs. Since significant progress has been made on the structural bases of CK2 inhibition, it is now possible to develop potent and selective cell-permeable CK2 inhibitors [29], [30]. Understanding the difference in sequences of the *CSNK2A1P* gene polymorphisms may allow us to design specific diagnostic tests for human cancer. Importantly, our results support the notion that protein kinase CK2α is an appealing target for cancer therapeutics such as small-molecule inhibitors [10], [31].

## Materials and Methods

### Cell lines

The human cancer (Jakurt, H1299, A549, A427, H441, H1703, H322, H460, HCT116, H1975, H322, H358, H838, H28, H2052, Hela and H1650) and normal lung (WI-38 and CCL-211) cell lines were obtained from American Type Culture Collections (Manassas, VA). H290 and MS-1 cell lines were obtained from NIH (Frederick, MD). Cells were grown in complete growth medium (Dulbecco's modified Eagle's medium for HeLa, A549 and CCL-211; Eagle's Minimum Essential Medium for WI-38 ; Roswell Park Memorial Institute's medium for H1299, A549, A427, H441, H1703, H322, H460, HCT116, H1975, H322, H358, H838, H28, H2052 and H1650) supplemented with 10% fetal bovine serum, 10 units/ml penicillin and 10 µg/ml streptomycin at 37°C and 5% CO2.

### Tissue samples

Fresh tumor tissues and adjacent normal tissues were obtained from patients with non-small cell lung cancer (NSLC) who were undergoing surgical resection of the primary tumor. The study was approved by the University of California, San Francisco, institutional review board (CHR# H8714-11647-14). We obtained written informed consents from all participants involved in our study. Tissue samples were kept at −180°C liquid nitrogen freezers before use, and final pathologic diagnosis was confirmed by a pathologist at the University of California, San Francisco (UCSF), USA. Normal adult genomic DNA form peripheral blood was purchased from BioChain (Hayward, CA).

### Fluorescence-in situ hybridization

The fluorescence-in situ hybridization (FISH) probe for CSNK2A1P (RP11-567I13, chromosome 11p15.3) was purchased from BACPAC Resources (Oakland, CA). The chromosome 11 centromere was labeled by Vysis CEP 11 SpectrumGreen™ probe (Abbott Molecular, Abbott Park, IL). Metaphase slides were prepared using standard protocols of the UCSF Molecular Pathology Core facility. All hybridizations were done by the UCSF Molecular Pathology Core facility. The CSNK2A1P BAC probe was labeled with Cy3 red by nick translation. Probe mixture was prepared according to the standard protocol.

### DNA and cDNA sequencing analysis

Genomic DNA or total RNA was isolated from cell lines and tissue samples using the DNeasy Blood & Tissue Kit or the RNeasy Mini Kit (Qiagen Valencia,CA), respectively. The CSNK2A1P gene was PCR amplified using its gene-specific primers. The forward and reverse primers used for PCR and sequencing were: 5′-AGAAAATTGCTCCCCACTCC-3′ and 5′-GTGCTGCCAGAGAATGA CAA-3′ respectively. The PCR products were gel-purified using the QIAquick Gel Extraction kit (Qiagen Valencia,CA) and were subsequently sequenced at MCLab (South San Francisco, CA).

### Semi-quantitative reverse transcription-PCR (RT-PCR) analysis

Total RNA from cell lines and tissues was isolated using an extraction kit, and DNA was eliminated by on-column treatment with DNase (RNeasy Mini kit; Qiagen, Valencia, CA, USA). Semiquantitative RT-PCR was performed by using SuperScript One-step RT-PCR with Platinum Taq kit (Invitrogen, Carlsbad, CA) according to the manufacturer's protocol. One-step RT-PCR was performed using pairs of CSNK2A1P-specific primers (Forward: 5′-AGAAAATTGCTCC CCACTCC-3′ and Reverse: 5′-GTGCTGCCAGAGA ATGACAA-3′), CSNK2A1-specific primers (Forward: 5′-TGGGGACAGAAGATTTATATGA-3′ and Reverse: 5′-CTGAAGAAATCCCTGACA TCAT-3′) and CSNK2B-specific primers (Forward: 5′-CAGGTCCCTCACTACCGACA -3′ and Reverse: 5′-CAGCTGGTAGGCCATCGGAT-3′). The PCR products were verified by direct DNA sequencing. Glyceraldehyde-3-phosphatedehydrogenase (GAPDH) was used as an internal control. Amplification conditions for CSNK2A1P, CSNK2A1 and CSNK2B were as follows: 1 cycle of 45°C for 30 min, followed by 1 cycle of 95°C for 5 min, and 35 cycles of 95°C for 1 min, 56°C for 1 min, 72°C for 1 min and 1 cycle of 72°C for 10 min then 4°C.

### Cloning of CSNK2A1P cDNAs

The full-length cDNA of CSNK2A1P gene from A549 cells was cloned using TOPO TA Cloning Kit (Invitrogen Carlsbad, CA), and then was subcloned into the HindIII and BamHI sites of the pcDNA3.1/myc-His vector (Invitrogen, Carlsbad, CA). The forward and reverse primers used for cloning were 5′-CCTTAAAAGCTTGACCATGTCGG GACCCGTGCCAAG-3′ and 5′-CCTTAAGGA TCCGACTGCTGAGCGCCAGCGGCAG-3′′respectively. The CSNK2 A1pcDNA3.1/myc-His vector was a gift from Dr. L. A. Pinna. Both myc-His tagged CSNK2A1 and CSNK2A1P genes were subsequently cloned into the SnaBI site of the pBabe-puro vector. The forward and reverse primers used for cloning were 5′-GGCTAGTTTACGTAG ACCATG TCGGGACCCGTG CCAAG-3′ and 5′-AAGGCACAGT CGACGCT GATCAGCGGGTT TAAACTCA-3′ respectively. All resultant vectors were verified by direct DNA sequencing.

### Retroviral production and transduction

The CSNK2A1 and CSNK2A1P retroviral vectors were then transfected into the HEK 293 Phoenix ampho packaging cells (ATCC, Manassas, VA) by using Fu-GENE6 transfection reagent (Roche, Lewes, UK) to produce retroviral supernatants. Forty-eight hours after transfection, the supernatant was filtered through a 0.45 µm syringe filter. Retroviral infection was performed by adding filtered supernatant to mesothelioma cell lines cultured on 10cm dishes with 50% confluent in the presence 8 ug/ml of polybrene (Sigma, St. Louis, MO). Six hours after infection, the culture medium was replaced with fresh medium and infected cells were allowed to recover for 48 hours. Infected cells were selected by adding 1µg/ml puromycin (Sigma, St. Louis, MO) to the culture medium for 48 hours and then maintained in complete medium with 0.5µg/ml puromycin. Empty retroviral infected stable cell lines were also produced by the above protocols.

### Western blot analysis

Whole protein was extracted by M-PER Mammalian Protein Extraction Reagent from cell lines added with Phosphatase Inhibitor Cocktail Set II (Calbiochem, San Diego, CA) and Complete Protease Inhibitor Cocktails (Roche, Lewes, UK) according to manufactures' protocols. The proteins were separated on 4–15% gradient SDS–polyacrylamide gels and transferred to Immobilon-P membranes (Millipore, Bellerica, MA). The following primary antibodies were used: anti-CK2α, anti-β-actin (Sigma Chemical, St. Louis, MO), anti-PML (Santa Cruz, Santa Cruz, CA), and anti-Myc tag (Cell Signaling, Danvers, MA). After being incubated with appropriate secondary antibodies, the antigen-antibody complexes were detected by using an ECL blotting analysis system (Amersham Pharmacia Biotech, Piscataway, NJ).

### Colony formation assay

NIH3T3 cells stably transfected with CSNK2A1 or CSNK2A1P (5×10^2^) were plated in 10 cm culture dishes and incubated in complete medium for 14 days. The colonies were then stained with 0.1% crystal violet, and colonies greater then 50 cells were counted. Results were expressed as relative colony formation: percentage of the number of colonies relative to the empty vector transfected controls. Three independent experiments were performed.

### Soft agar growth assay

For experiments using the pcDNA3.1/myc-His vectors expressing CSNK2A1 and CSNK2A1P, NIH3T3 cells were transfected with these vectors or control empty vector and then selected in 100 ug/ml of G418 for 1 week. Cells (1×10^3^) were then cultured in DMEM plus 15% FBS in 0.35% (w/v) low melting temperature agar between layers of 0.7% low melting temperature agar. After 4 weeks, colonies were stained with 3-(4, 5-dimethylthiazol-2-yl)-2,5-diphenyltetrazolium bromide (Sigma Chemical Co.), and colonies containing >100 cells were scored. Colonies were photographed and counted after staining.

### Protein degradation assay

The stable NIH3T3 cells retrovirally transfected with CSNK2A1 and CSNK2A1P were plated on 6 cm culture dishes. At 80% confluence, cells were exposed to 20 µg/ml cycloheximide and harvested at different time points (0, 2 and 6 hours). Total cellular proteins were extracted and were analyzed by western blot analysis using β-actin as the loading control.

### Co-immunoprecipitation assay

293T cells were transiently co-transfected with PML pcDNA4/V5-His (a gift from Dr. Zheng Pan) and CSNK2A1 or CSNK2A1P pcDNA3.1/myc-His vectors with Lipofectamine 2000 transfection reagent (Invitrogen Carlsbad, CA). Twenty-four hours after transfection, cells were treated with 10µM of MG132 (Sigma, St. Louis, MO) and then harvested in NP-40 lysis buffer (150 mM NaCl, 50 mM Tris [pH 8.0], 1% NP40), protease inhibitor, and phosphatase inhibitor cocktail (Roche, Lewes, UK). Immunoprecipitation was performed by the Catch and Release v2.0 Reversible Immunoprecipitation System (Millipore, Bellerica, MA) according to the manufacturer's protocols. Anti-Myc tag (Santa Cruz, Santa Cruz, CA) and anti-V5 tag (Invitrogen, Carlsbad, CA) antibodies were used for immunoprecipitation respectively.

### Transfection of small interfering RNA

Pre-designed and validated *CSNK2A1P* and universal negative control small interfering RNAs (siRNA) were purchased from Invitrogen (Carlsbad, CA). Transfection was performed using Lipofectamine™ RNAiMAX Transfection Reagent (Invitrogen), according to the manufacturer's manual. Cells were plated in 60-mm dishes in antibiotic-free media and transfection was performed with cells at 60% confluence with a final concentration of 50 nM for each siRNA. At 72 hours after transfection, cells were analyzed for gene and protein expression.

### Allele-specific amplification assays

The allele-specific amplification was measured using the ABI PRISM 7700 sequence detection system. PCR reactions for allele-specific expression (5 µl) contained 10 ng genomic DNA, 1× TaqMan universal PCR master mix, forward and reverse primers (900 nM), 200 nM VIC-labeled probe and 200 nM FAM-labeled probe. Amplification conditions were as follows: 1 cycle of 95°C for 10 min, followed by 40 cycles of 95°C for 15 s and 63°C for 1 min. The data was analyzed using the Allelic Discrimination Sequence Detection Software (Applied Biosystems). The TaqMan primers and probes were custom designed using the Primer Express Oligo Design Software (Applied Biosystems). Probes were MGB probes were designed specifically for TaqMan Allelic Discrimination (Applied Biosystems). Primer sequences are 5′-CCGCCTTGGTTTTTGAACAC-3′and 5′-GGCCTTCAGAATCTCATACATGTAAA-3′; probe sequences are FAM-CACAGACTATGACTC (398C allele specific) and VIC-TCACAGACTATGATTC (398T allele specific). PCR was done in triplicate for each sample and experiments were repeated at least three times. CT values were normalized to the average normal genomic CT difference in each experiment. The CT value differences between the two probes for the triplicates were then averaged.

### Kinase assays

For determination of the kinase activity of the expressed CSNK2A1 and CSNK2A1P proteins, Casein Kinase 2 Assay Kit (Millipore, Bedford, MA) was used according to manufacture's protocol. Stable NIH3T3 cells transfected with the CSNK2A1 and CSNK2A1P genes were harvested in NP-40 lysis buffer (150 mM NaCl, 50 mM Tris [pH 8.0], 1% NP40), protease inhibitor, and phosphatase inhibitor cocktail (Roche, Lewes, UK). Immunoprecipitation was performed by the Catch and Release v2.0 Reversible Immunoprecipitation System (Millipore, Bellerica, MA) according to the manufacturer's protocols. Anti-Myc tag (Santa Cruz, Santa Cruz, CA) antibody was used for immunoprecipitation. Kinase assay was carried in a final volume of 50 µl containing 20 mM MOPS (pH 7.2), 25 mM β-glycerol phosphate, 5 mM EGTA, 1 mM sodium orthovanadate, 1 mM dithiothreitol, 15 mM Mgcl_2_, 200 µM for CK2 substrate peptide: RRRDDDSDDD (Millipore, Bedford, MA), and [γ-33P]-ATP. After incubation in 30°C for 20 minutes, assay was stopped by adding of 20 µl 4% trichloroacetic acid and transferred 25 µl to P81 phosphocellulose squares. After washing with 0.75% phosphoric acid for 6 times and with acetone for 1 time, phosphocellulose squares were dried and transferred to scintillation vials for counting.

### Statistical analysis

The data are shown as mean values ± standard deviation (SD). Student's *t*-test was used to compare results between control and experimental groups in the colony formation assay. Chi-square test was used to compare the frequency of the *CSNK2A1P* polymorphisms between lung cancer tissues and normal controls. Pearson correlation coefficient was used to access the correlation between mRNA expression and copy numbers of the *CSNK2A1P* gene in cancer cell lines. Statistical analysis was carried out using SPSS (version 10.0, Chicago, IL). A P value of less than 0.05 was considered statistically significant. All statistical tests were two-sided.

## Supporting Information

Figure S1 Alignment of primers for semi-quantitative RT-PCR of the *CSNK2A1P* (F1 and R1) and *CSNK2A1* (F2 and R2) genes. Primers used for PCR and DNA sequencing (F1 and R3) were also shown. Open reading frames of both genes were represented by the solid bars. Regions specific to the *CSNK2A1P* gene was represented as the thin hollow bar. The vertical arrows indicate the differences of the sequences between the *CSNK2A1* and *CSNK2A1P* genes used for designing of primers. F1: 5′-AGAAAATTGCTCC CCACTCC-3′. R1: 5′-GTGCTGCCAGAGA ATGACAA-3′), F2: 5′-TGGGGACAGAAGATTTATATGA-3′. R2: 5′-CTGAAGAAATCCCTGACA TCAT-3′). R3: 5′-GTGCTGCCAGAGAATGA CAA-3.
(0.05 MB DOC)
