# Supplementary material for: Functional Polymorphism of the CK2α Intronless Gene Plays Oncogenic Roles in Lung Cancer
Source: PLoS One. 2010 Jul 2;5(7):e11418. doi: 10.1371/journal.pone.0011418 (PMC2896393; doi:10.1371/journal.pone.0011418)
Supplement: Figure S1 — Alignment of primers for semi-quantitative RT-PCR of the CSNK2A1P (F1 and R1) and CSNK2A1 (F2 and R2) genes. Primers used for PCR and DNA sequencing (F1 and R3) were also shown. Open reading frames of both genes were represented by the solid bars. Regions specific to the CSNK2A1P gene was represented as the thin hollow bar. The vertical arrows indicate the differences of the sequences between the CSNK2A1 and CSNK2A1P genes used for designing of primers. F1: 5′-AGAAAATTGCTCC CCACTCC-3′. R1: 5′-GTGCTGCCAGAGA ATGACAA-3′), F2: 5′-TGGGGACAGAAGATTTATATGA-3′. R2: 5′-CTGAAGAAATCCCTGACA TCAT-3′). R3: 5′-GTGCTGCCAGAGAATGA CAA-3. (0.05 MB DOC) [file pone.0011418.s001.doc]

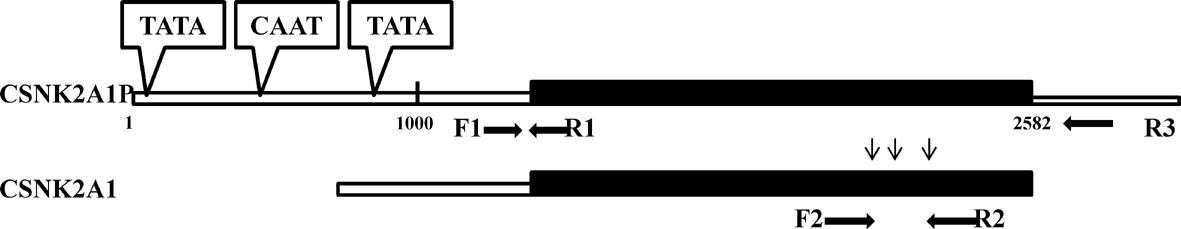


FigureS1. Alignment of primers for semi-quantitative RT-PCR of the *CSNK2A1P* (F1 and R1) and *CSNK2A1* (F2 and R2) genes. Primers used for PCR and DNA sequencing (F1 and R3) were also shown. Open reading frames of both genes were represented by the solid bars. Regions specific to the *CSNK2A1P* gene was represented as the thin hollow bar. The vertical arrows indicate the differences of the sequences between the *CSNK2A1* and *CSNK2A1P* genes used for designing of primers. F1: 5’-AGAAAATTGCTCC CCACTCC-3’. R1: 5’-GTGCTGCCAGAGA ATGACAA-3’), F2: 5’-TGGGGACAGAAGATTTATATGA-3’. R2: 5’-CTGAAGAAATCCCTGACA TCAT-3’). R3: 5'-GTGCTGCCAGAGAATGA CAA-3.
